# Analysis of characteristics of movement disorders in patients with anti-N-methyl-D-aspartate receptor encephalitis

**DOI:** 10.3389/fneur.2024.1357697

**Published:** 2024-03-01

**Authors:** Hongmei Li, Jiajie Chen, Pinyi Zhou, Qiang Meng

**Affiliations:** ^1^Department of Neurology, The First People’s Hospital of Yunnan Province, The Affiliated Hospital of Kunming University of Science and Technology, Kunming, China; ^2^Department of Sleep Medicine, The First People’s Hospital of Yunnan Province, The Affiliated Hospital of Kunming University of Science and Technology, Kunming, China

**Keywords:** anti-NMDAR encephalitis, clinical characteristic, movement disorders, hyperkinetic, severity of disease, prognosis

## Abstract

**Objective:**

Movement disorders (MDs) are common in anti-N-methyl-D-aspartate receptor (NMDAR) encephalitis but are poorly studied. This study aimed to investigate the clinical characteristics of MDs and the clinical differences between patients with and without MDs in anti-NMDAR encephalitis.

**Methods:**

A retrospective study was conducted on patients with anti-NMDAR encephalitis who were first diagnosed and treated in the First People’s Hospital of Yunnan Province from January 2017 to September 2022. According to the presence or absence of MDs, all patients were divided into two groups, and the clinical manifestations, auxiliary examinations, and prognosis of the two groups were compared. Patients in the MDs group were further subgrouped by different ages (<12 years, 12–17 years, and ≥ 18 years) and genders, and the prevalence of each MD was compared in different age and gender groups.

**Results:**

(1) In our study there were 64 patients, of whom 76.6% (49/64) presented with MDs; the median age of onset in patients with MDs was 21 (15,35) years and 65.3% (32/49) were female. The three most common MDs were orofacial dyskinesia (OFLD) (67.3%), dystonia (55.1%), and stereotypies (34.7%). Patients <12 years were more likely to experience chorea than patients in other age groups (*p* = 0.003). (2) Compared with the non-MDs group, patients in the MDs group showed higher rates of prodromal manifestations, autonomic dysfunction, consciousness disorders, as well as pulmonary infection and gastrointestinal dysfunction (all *p* < 0.05). Peripheral blood neutrophil to lymphocyte ratio (NLR) (*p* = 0.014), the proportion of cerebrospinal fluid (CSF) NMDAR antibody titers ≥1:32 (*p* = 0.047), ICU admission rate (*p* = 0.04), length of stay (*p* = 0.007), maximum mRS score in the course of disease (*p* = 0.001) and mRS score at discharge (*p* = 0.006) in the MDs group were significantly higher than the non-MDs group.

**Conclusion:**

MDs associated with anti-NMDAR encephalitis were predominantly hyperkinetic. Chorea occurred more commonly in patients aged <12 years. Patients with MDs were prone to autonomic dysfunction, consciousness disorders, pulmonary infection, and gastrointestinal dysfunction; they had more intense inflammation, more severe disease, and a poorer short-term prognosis.

## 1 Introduction

Anti-N-methyl-D-aspartate receptor (NMDAR) encephalitis is an autoimmune disease of the central nervous system mediated by GluN1 subunit antibodies of the NMDAR and is the most common type of autoimmune encephalitis (1). Its main clinical symptoms include psychiatric symptoms, cognitive disorders, epilepsy, movement disorders (MDs), etc. (1). MDs are common in anti-NMDAR encephalitis with a certain specificity and can assist in the diagnosis of the disease. However, due to the diversity and complexity of this symptom, misdiagnosis of the disease can also result. Moreover, persistent unrelieved MDs may affect patients’ quality of survival and even be life-threatening (2–4). Therefore, early recognition and diagnosis of MDs are particularly important. Yet, clinical awareness and attention to this symptom are currently insufficient, research on the specific features of MDs associated with anti-NMDAR encephalitis is inadequate, and there is a lack of studies exploring the clinical factors associated with this symptom.

In the present study, we aimed to analyze the clinical characteristics of MDs associated with anti-NMDAR encephalitis, and to explore the potential association between MDs and clinical features, disease severity, and prognosis, to improve clinicians’ awareness of the symptoms, to achieve early diagnosis and treatment of the disease, and to better improve the patients’ conditions and prognosis; it also helps to better understand the mechanism of the disease, and provides new ideas for the diagnosis and treatment of anti-NMDAR encephalitis.

## 2 Materials and methods

### 2.1 Study subjects

Retrospective analysis of the clinical data of patients with anti-NMDAR encephalitis admitted to the First People’s Hospital of Yunnan Province from January 2017 to September 2022. Inclusion criteria (5) were as follows: (1) meeting one or more of the following major symptoms after reasonable exclusion of other diseases: abnormal psychiatric behavior or cognitive dysfunction, speech impairment, seizures, MDs/involuntary movements, consciousness disorders, autonomic dysfunction or central hypoventilation; (2) cerebrospinal fluid (CSF) positive for anti-NMDA receptor antibodies. Exclusion criteria: (1) patients lacking key data; (2) anti-NMDAR encephalitis was confirmed and treated in other healthcare centers before admission.

### 2.2 Methods

The following clinical data of enrolled patients were collected by querying the electronic medical record system, including age, sex, prodromal symptoms, first symptoms, main clinical manifestations, complications during hospitalization, presence of ovarian teratoma, immunotherapy, days of hospitalization, intensive care unit (ICU) admission, cranial brain magnetic resonance imaging (MRI), electroencephalogram (EEG), initial peripheral serology and CSF examinations on admission, CSF NMDAR antibody titers. The modified Rankin scale (mRS) was used to assess the neurological function of all patients (6), maximum mRS scores during the disease and at discharge were recorded separately to evaluate the severity and short-term prognosis.

The prodromal symptoms referred to the symptoms that precede the main clinical manifestations of anti-NMDAR encephalitis, including headache, fever, and tiredness, etc. The first symptoms were defined as the main clinical manifestations of the patient at the beginning of the disease. The main clinical manifestations referred to the main clinical symptoms in the diagnostic criteria for anti-NMDAR encephalitis: including abnormal psychiatric behavior or cognitive dysfunction, speech impairment, seizures, MDs/involuntary movements, consciousness disorders, autonomic dysfunction. Peripheral serum examination included white blood cell (WBC) count, neutrophil count, lymphocyte count, and neutrophil-lymphocytes ratio (NLR). CSF examination included NMDAR antibody titers (≥ 1:32 for high titers, < 1:32 for low titers), WBC count (> 5 × 10^6^/L for increased WBC count), and protein quantification (> 450 mg/L for elevated protein quantification). Abnormal cranial MRI findings were defined as abnormal signals on T2/T2-fluid attenuated inversion recovery or enhancement sequences, and the sites of abnormal signals were recorded and roughly classified into the limbic, basal ganglia, and cortex. The digital multifunctional EEG was used to monitor the patient’s EEG using the International 10–20 System Scalp EEG placement for EEG monitoring of patients. Short-range or long-range EEG data were collected from all patients at the onset of illness, epilepsy, and movement disorders, to record whether the EEG manifestations were normal or not, and to classify abnormal EEG manifestations broadly into slow waves and epileptic waves. Differentiation by bedside video EEG findings if seizures were difficult to distinguish from MDs. The patients were divided into two groups according to the presence or absence of MDs during the disease: one with MDs and another without, and the above clinical data were compared between the two groups.

Referring to previous studies, this study classified specific symptoms of MDs as orofacial dyskinesia (OFLD), stereotypies, dystonia, myoclonus, tremor, catatonia, ataxia, muscle weakness, and chorea (defined in the Supplemental materials) (7–9). Based on the previous hypothesis that the degree of dopamine receptor maturation may be related to different MDs, the patients in the MDs group were divided into three age groups: < 12 years old, 12–17 years old, and ≥ 18 years old, and the occurrence of different MDs in different age groups was compared. In addition, patients with MDs were grouped into males and females, and the incidence of different MDs was compared between the different gender groups.

This study was approved by the Research Ethics Committee of the First People’s Hospital of Yunnan Province and carried out in compliance with the Declaration of Helsinki.

### 2.3 Statistical analysis

SPSS 22.0 was applied for statistical analysis. The continuous variables with normal distribution were expressed as means ± standard deviations, and the independent t-test was used for data comparisons between groups; the continuous variables without normal distribution were expressed as medians (interquartile ranges, IQR) and the Mann–Whitney U test was employed for intergroup comparisons of data. The categorical variables were expressed as frequency (percentage) and compared using the Chi-square test, continuous correction Chi-square test, or Fisher’s exact probability method. The *value of p* <0.05 was considered statistically significant.

## 3 Results

### 3.1 Specific symptoms and incidence of MDs

**Figure 1:**
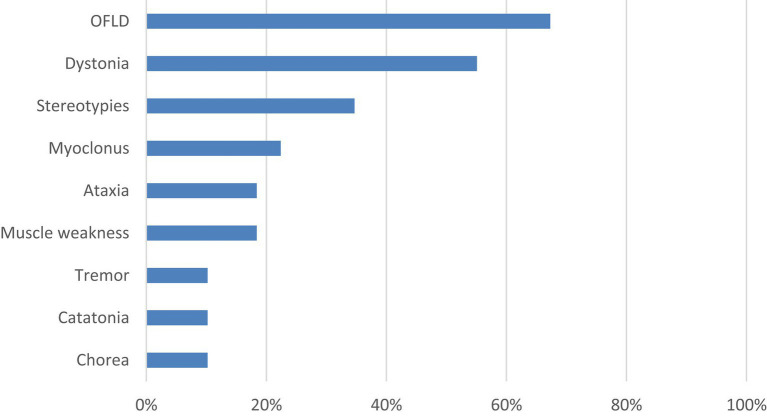
Specific symptoms and incidence of MDs. MDs, movement disorders; OFLD, orofacial dyskinesia.

A total of 64 patients were included in the study, including 24 (37.5%) males and 40 (62.5%) females, with a median age of 24 (15,38) years. There were 49 patients (76.6%) with MDs, among which 37 patients (75.5%) manifested at least two types of MDs, specific symptoms including OFLD (33 patients, 67.3%), dystonia (27 patients, 55.1%), stereotypies (17 patients, 34.7%), myoclonus (11 patients, 22.4%), ataxia (9 patients, 18.4%), muscle weakness (9 patients, 18.4%), tremor (5 patients, 10.2%), catatonia (5 patients, 10.2%), and chorea (5 patients, 10.2%). The prevalence and category of all MDs are detailed in Figure 1 and Supplementary Table S1.

**Figure 2:**
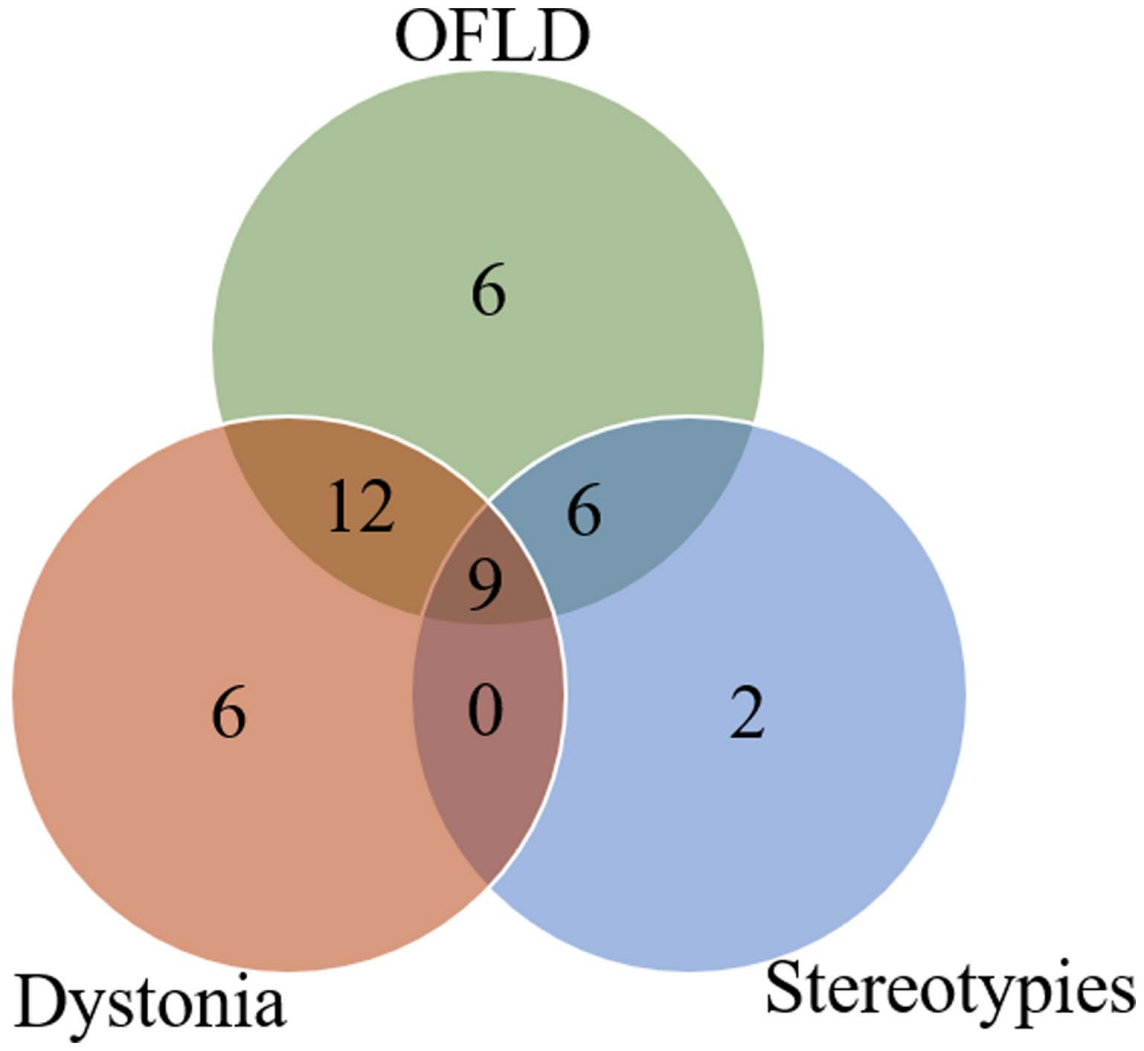
Intersection analysis between OFLD, stereotypies and dystonia in anti-NMDAR encephalitis. OFLD, orofacial dyskinesia.

In addition, the intersection analysis between the three most common types of MDs is shown in Figure 2. It was found that the number of patients who experienced OFLD, dystonia, and stereotypies during the course of the disease (*n* = 9) exceeded those who appeared OFLD (*n* = 6), dystonia (*n* = 6), or stereotypies (*n* = 2) in isolation.

### 3.2 Comparison of the occurrence of different MDs in different age and gender groups

**Figure 3:**
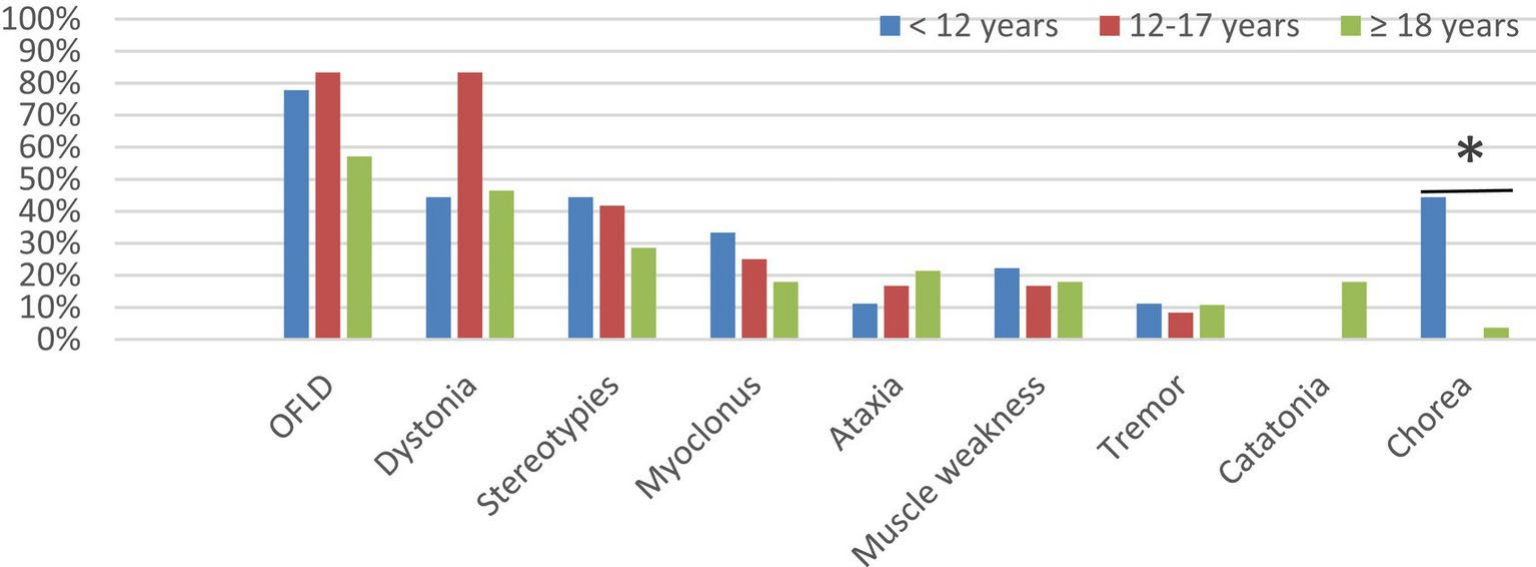
Comparison of the incidence of different MDs in different age groups. MDs, movement disorders; OFLD, orofacial dyskinesia. * Means the difference was statistically significant compared to the age < 12 years group, *p* = 0.003.

All patients <12 years in this study developed MDs; 80.0% (12/15) of patients aged 12–17 years experienced MDs; and 70.0% (28/40) of patients aged ≥18 years experienced MDs. To explore the potential association between age and different MDs in anti-NMDAR encephalitis, patients in the MDs group were further grouped by age: 9 patients <12 years, 12 patients 12–17 years, 28 patients ≥18 years. As shown in Figure 3 and Supplementary Table S2, the commonest MD in each age group was OFLD, followed by dystonia and stereotypies. Chorea was more common in patients aged <12 years than in the other two groups (*p* = 0.003). Catatonia was present only in patients aged ≥18 years, but it as well as other MDs did not differ significantly between age groups (*p* > 0.05).

**Figure 4:**
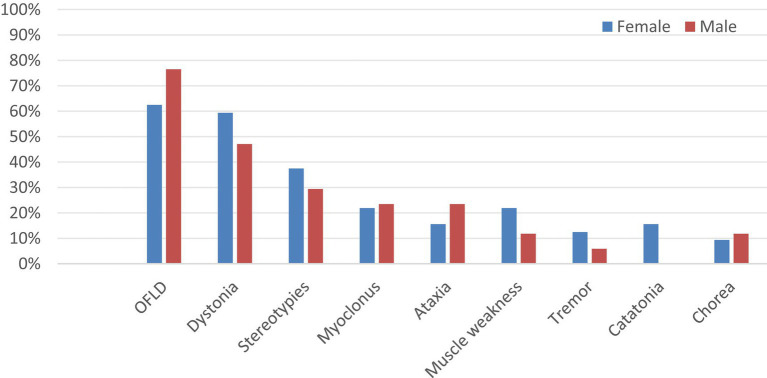
Comparison of the incidence of different MDs in different gender groups. MDs, movement disorders; OFLD, orofacial dyskinesia.

There were 40 female and 24 male patients in this study and the prevalence of MDs was 80.0 and 70.8% in female and male patients, respectively. To explore the potential association between gender and different MDs in anti-NMDAR encephalitis, the patients in the MDs group were further grouped by gender: 32 (65.3%) patients were female, and 17 (34.7%) were male. As shown in Figure 4 and Supplementary Table S3, the three most common dyskinesia manifestations in male and female patients remained as follows OFLD, dystonia, and stereotypes. Statistical analysis showed that there was no statistically significant difference between the types of MDs in patients of different genders (*p* > 0.05).

### 3.3 Comparison of the clinical data in the MDs and non-MDs groups

The comparison of clinical data between the two groups is shown in Table 1. In terms of demographic characteristics, the proportion of female patients in the MDs group was slightly higher than in the non-MDs group (65.3% vs. 53.3%) and the median age at onset was lower than in the non-MDs group [21 (15, 35) years vs. 30 (22, 43) years], but no significant differences were observed between the two groups in terms of age and sex (*p* > 0.05). Regarding the clinical manifestations, patients in the MDs group than those in the non-MDs group showed higher rates of prodromal symptoms (71.4% vs. 40.0%, *p* = 0.026), consciousness disorders (69.4% vs. 40.0%, *p* = 0.04), autonomic dysfunction (59.2% vs. 20.0%, *p* = 0.008), pulmonary infections (49.0% vs. 20.0%, *p* = 0.047), and gastrointestinal dysfunction (38.8% vs. 6.7%, *p* = 0.042). The remaining clinical symptoms and complications were not significantly different between the two groups.

**Table 1:** Comparison of clinical data between two groups of patients with or without MDs.

Variable	MDs group (*n* = 49)	Non-MDs group (*n* = 15)	*P*
Sex, female *n* (%)	32 (65.3)	8 (53.3)	0.402
Age, median (IQR), year	21 (15, 35)	30 (22, 43)	0.088
Previous encephalitis history, *n* (%)	8 (16.3)	4 (26.7)	0.603
Prodromal symptoms, *n* (%)	35 (71.4)	6 (40.0)	0.026
Headache	16 (32.7)	5 (33.3)	1.000
Fever	15 (30.6)	4 (26.7)	1.000
Tiredness	11 (22.4)	1 (6.7)	0.321
First symptoms, *n* (%)			
Mental and behavioral abnormalities	26 (53.1)	4 (26.7)	0.073
Epilepsy	13 (26.5)	6 (40.0)	0.499
Cognitive impairment	2 (4.1)	2 (13.3)	0.232
Main clinical manifestations, *n* (%)			
Mental and behavioral abnormalities	45 (91.8)	13 (86.7)	0.618
Consciousness disorders	34 (69.4)	6 (40.0)	0.040
Epilepsy	30 (61.2)	7 (46.7)	0.318
Sleep disturbances	29 (59.2)	8 (53.3)	0.668
Autonomic dysfunction	29 (59.2)	3 (20.0)	0.008
Cognitive impairment	23 (46.9)	7 (46.7)	0.840
Speech disturbances	20 (40.8)	3 (20.0)	0.142
Complications, *n* (%)			
Pulmonary infection	24 (49.0)	3 (20.0)	0.047
Abnormal liver function	30 (61.2)	7 (46.7)	0.318
Electrolyte metabolism disorders	24 (49.0)	4 (26.7)	0.127
Gastrointestinal dysfunction	19 (38.8)	1 (6.7)	0.042
Urinary tract infection	15 (30.6)	2 (13.3)	0.321
Respiratory failure	13 (26.5)	1 (6.7)	0.204
Ovarian teratoma, n (%)	1 (2.0)	1 (6.7)	0.417
Abnormal brain MRI, n (%)	25 (51.0)	7 (46.7)	0.768
Cerebral cortex	19 (38.8)	6 (40.0)	0.932
Limbic	18 (36.7)	6 (40.0)	0.819
Basal ganglia	6 (12.2)	2 (13.3)	1.000
Abnormal EEG, *n* (%)	43 (87.8)	10 (66.7)	0.133
Slow waves	38 (77.6)	9 (60.0)	0.311
Epileptic discharge	14 (28.6)	3 (20.0)	0.746
CSF routine tests, n (%)			
Increased WBC count	31 (64.6)	7 (46.7)	0.216
Increased protein in CSF	8 (16.3)	4 (26.7)	0.603
CSF NMDAR antibody titers, *n* (%)			0.047
<1:32	25 (51.0)	12 (80.0)	
≥1:32	24 (49.0)	3 (20.0)	
Overlap other antibodies	5 (10.2)	2 (13.3)	1.000
Blood tests, median (IQR)			
WBC, ×10^9^/L	9.00 (5.87, 11.53)	7.19 (5.96, 9.42)	0.471
Neutrophil, ×10^9^/L	6.63 (4.27, 9.57)	5.29 (3.35, 6.32)	0.161
Lymphocyte, ×10^9^/L	1.10 (0.88, 1.85)	1.97 (1.50, 2.33)	0.034
NLR	5.30 (2.59, 10.27)	2.71 (2.23, 3.46)	0.014
First line immunotherapy, *n* (%)			0.863
Steroids	7 (14.3)	3 (20.0)	
Steroids +IVIg	39 (79.6)	11 (73.3)	
Steroids +IVIg +Plasma exchange	3 (6.1)	1 (6.7)	
Second line immunotherapy, *n* (%)	9 (18.4)	2 (13.3)	0.951
ICU admission, *n* (%)	34 (69.4)	6 (40.0)	0.040
Hospital days, median (IQR)	25.0 (15.0, 33.5)	14.0 (12.0, 16.0)	0.007
mRS score, median (IQR)			
Maximum mRS score in the course of disease	4.0 (3.5–5.0)	3.0 (2.0–4.0)	0.001
Discharge mRS score	3.0 (2.5–4.0)	2.0 (1.0–3.0)	0.006

MDs, movement disorders; MRI, magnetic resonance imaging; EEG, electroencephalogram; CSF, cerebrospinal fluid; WBC, white blood cell count; NMDAR, N-methyl-D-aspartate receptor; NLR, neutrophil-lymphocytes ratio; IVIg, intravenous immunoglobulins; ICU, Intensive Care Unit; mRS, modified Rankin scale.

Compared to patients without MDs, those with MDs had higher peripheral blood NLR [5.30 (2.59, 10.27) vs. 2.71 (2.23, 3.46), *p* = 0.014], and the percentage of CSF NMDAR antibody titers ≥1:32 (49% vs. 20%, *p* = 0.047). In addition, patients in the MDs group had higher ICU admission rate (69.4% vs. 40.0%, *p* = 0.040) and longer days of hospitalization [25.0 (15.0, 33.5) vs. 14.0 (12.0, 16.0), *p* = 0.007]; their maximum mRS during the disease [4.0 (3.5, 5.0) vs. 3.0 (2.0, 4.0), *p* = 0.001] and mRS at discharge [3.0 (2.5, 4.0) vs. 2.0 (1.0, 3.0), *p* = 0.006] were significantly greater than the non-MDs group, indicating that they had more severe disease and worse short-term prognosis. The remaining CSF examination, abnormal cranial MRI rate, and EEG performance did not find significant differences between the two groups (all *p* > 0.05).

## 4 Discussion

This study investigated the characteristics of MDs in anti-NMDAR encephalitis and analyzed the differences in clinical characteristics of patients with and without MDs. Our research found that (1) anti-NMDAR encephalitis is often combined with multiple MDs, with OFLD, dystonia, and stereotypies being the most common, and pediatric patients <12 years are more likely to develop chorea than older patients; (2) patients with MDs in anti-NMDAR encephalitis have a higher incidence of consciousness disorders, autonomic dysfunction, pulmonary infection, and gastrointestinal dysfunction during the disease, and a higher NLR and CSF NMDAR antibody titers; they tend to be more severe and have a relatively poor prognosis.

More than half of the patients with anti-NMDAR encephalitis in our study (76.6%) had MDs, of whom 75.5% could present with two or more manifestations of MDs, with hyperkinetic movements such as OFLD, dystonia, and stereotypies being the most common, which can involve multiple parts of the body and constitute complex and difficult to define movement symptoms, making the diagnosis difficult. However, the current diagnosis of MDs in anti-NMDAR encephalitis is mostly based on literature reports and clinical experience, lacking a systematic, standardized diagnostic consensus, which still needs further improvement.

According to the current study, MDs in anti-NMDAR encephalitis are mostly observed in female and pediatric patients (10, 11). Similar findings were found in our study, but sex and age of onset did not constitute a statistical difference between the two groups with and without MDs, which may be related to the small sample size of our study. With respect to sex, females have higher estrogen levels, research showed that sex hormones are involved in the regulation of the dopaminergic system and that estrogenic effects can cause or exacerbate hyperkinetic movements such as chorea, dystonia, and tics (12), which may explain the higher incidence of MDs in females with anti-NMDAR encephalitis than in males. Sex-related differences in MDs have not been adequately studied, with only a few literature reports and controversial conclusions. Therefore, it is essential to further investigate the role of sex hormones in the basal ganglia, sex differences in brain structure and function, and the interaction between genes and sex.

In terms of age, several studies have confirmed that the clinical manifestations of anti-NMDAR encephalitis vary among patients of different ages, adults mainly present with psychiatric and cognitive impairment, while MDs, seizures, and behavioral changes are more common in children (11, 13). In recent years, some scholars further explored the influence of age on the various MDs in anti-NMDAR encephalitis and found that hyperkinetic movements, such as chorea, were more often seen in younger patients, whereas hypokinetic movements, such as catatonia and bradykinesia, were more common in older patients (14, 15). Similar conclusions were reached in our study, with chorea being more common in pediatric patients aged <12 years than in older patients; furthermore, although the incidence of catatonia did not constitute a statistical difference between the different age groups, we observed catatonia only in adult patients. This may be related to the decrease of dopamine receptors with age and also to the distribution area and density of the distribution of dopamine receptors (14, 16).

In this study, a systematic review and analysis of the clinical data of patients in the MDs group and the non-MDs group revealed significant differences in clinical symptoms, complications, auxiliary examinations, disease severity, and short-term prognosis between the two groups.

The incidence of prodromal symptoms, consciousness disorders, and autonomic dysfunction was significantly higher in the MDs group. This may be related to the mechanism of MDs, where cross-linking and internalization of NMDA receptors mediated by NMDAR antibodies may lead to impaired glutamatergic transmission in inhibitory gamma-aminobutyric acid (GABA) neurons and diminished inhibition of the corpus striatum and limbic systems, causing disruption of the corticostriatal pathway and disinhibition of the brain stem, leading to involuntary movements (17). And the limbic system can also cause secondary autonomic dysfunction by affecting the autonomic centers of important visceral activities (18). The cerebral cortex and brainstem are involved in regulating the level of consciousness as well as motor function. As mentioned above, there is a complex link between MDs and consciousness-regulating brain regions and autonomic function-regulating centers, which may lead to a higher incidence of autonomic dysfunction and consciousness disorders in patients with MDs.

There is an association between MDs and epilepsy, which coexist or overlap in anti-NMDAR encephalitis (10, 19, 20). Recently, Hang et al. (10) studied 119 patients with anti-NMDAR encephalitis and found that patients with OFLD had a significantly higher incidence of epilepsy than those without OFLD (84.1% vs. 62.7%, *p* = 0.013). Our study showed that patients with MDs had a higher incidence of epilepsy than those without MDs (61.2% vs. 46.7%), but the results did not constitute a statistical difference. Clinically, it is customary to consider epilepsy as a disorder attributed to the cerebral cortex, and MDs mostly reflect dysfunction in subcortical areas. However, cortical and subcortical events should not be isolated due to the extensive and complex connections between the basal ganglia and the cerebral cortex, such as the frontal striatal pathway (19). For example, in a patient with both epilepsy and MDs, the epileptic source was identified in the motor cortex, but seizure phase discharges were also detected in the basal ganglia region (20). Therefore, the distinction between MDs and seizures can be determined by close clinical observation and video EEG monitoring, and the diagnostic boundaries and associations between the two remain to be revealed in subsequent studies.

It is worth mentioning that, our study observed 32 patients presented involuntary movements during sleep, such as chewing, smacking, hand groping, pedaling and myoclonus. Similar phenomena were mentioned in previous reports (21, 22), for which Stamelou et al. (22) proposed the hypothesis of status dissociatus, suggesting that involuntary movements occur in patients with severe sleep disturbances. The hypothesis is based on the highly similar clinical characteristics and pathophysiology between status dissociatus and anti-NMDAR encephalitis. NMDAR antibodies-mediated GABA dysfunction can cause frontostriatal syndrome and brainstem disinhibition (17), resulting in patients with MDs, autonomic dysfunction, and various sleep problems before onset, throughout the disease, and even after recovery (23). Therefore, video polysomnography in anti-NMDAR encephalitis seems important to explore the link between MDs and sleep disorders and to delve into the pathogenesis of anti-NMDAR encephalitis.

Patients with MDs in our study were more likely to develop pulmonary infections than patients without MDs. Miao et al.’s (24) study found that abnormal movements were an independent risk factor for the development of pneumonia (odds ratio = 3.716, 95% confidence interval = 1.149–12.015, *p* = 0.028). We consider the following reasons for the above finding may be as follows: firstly, OFLD involving the mouth, tongue, and face is the most common MD, which can affect the oral phase of the normal swallowing process (25), and may lead to dysphagia and aspiration. Secondly, patients with MDs are prone to concomitant consciousness disorders and autonomic dysfunction. The decreased level of consciousness can easily lead to prolonged bed rest, aspiration, or difficulty in respiratory secretion discharge, and autonomic nervous dysfunction may also induce or aggravate pulmonary infections (26, 27). Finally, the longer length of hospital stay in patients with MDs also increases the risk of infections.

In addition, we also observed that patients in the MDs group were prone to gastrointestinal dysfunction, possibly because they are prone to concomitant autonomic dysfunction, or perhaps because they may be treated with enteral nutrition in the presence of a reduced level of consciousness, OFLD, and other conditions that lead to restriction of autonomous eating, which increased the incidence of patients with diarrhea, constipation and other gastrointestinal dysfunction (28). Although other complications did not show significant differences in this study, overall, the incidence of each complication was slightly higher in the MDs group than in the non-MDs group. Therefore, patients with MDs should be closely monitored for complications and immunotherapy should be accompanied by aggressive symptomatic treatment.

Regarding cranial MRI findings, our study showed no significant difference in abnormal rates between the two groups. Another cohort follow-up study found that abnormal cranial MRI presentation in patients with anti-NMDAR encephalitis was not significantly correlated with clinical manifestations such as seizures, hypoventilation, and disturbance of consciousness (*p* > 0.05) (29). It may be because the mechanism of anti-NMDAR encephalitis is related to characteristic neuronal functional connectivity and extensive nerve fiber integrity changes, which are often not found on routine clinical MRI (30). In the future, more sensitive, multimodal, and multiparametric MRI techniques based on large samples are needed to further explore the functional brain status of patients with MDs in anti-NMDAR encephalitis and to understand the mechanisms of the disease from multiple perspectives.

In terms of peripheral serology and CSF findings, we found significant differences in NLR and CSF NMDAR antibody titers between the two groups. Peripheral blood NLR is a systemic inflammatory index and previous studies have suggested that its changes may reflect the changes in the balance between inflammatory and immune activity and may be used as the biological index to evaluate the severity and prognosis of patients with anti-NMDAR encephalitis (31–33). Our study showed that patients in the MDs group had higher NLR, suggesting that their inflammatory response was more intense, and indirectly pointing to a more severe condition and a poorer prognosis.

According to the study of Gresa-Arribas et al. (34), patients with poor prognosis had higher levels of CSF NMDAR antibodies expression. In our study, patients in the MDs group had higher titers of NMDAR antibodies, which can be inferred that they have a poorer prognosis. However, no studies explore the relationship between NMDAR antibody titers and the potential mechanisms of MDs. We speculate that it may be related to the extensive distribution of NMDAR antibodies in the cortex and striatum, and the specific mechanism needs to be further explored.

On the severity and prognosis, our study showed that patients with MDs had longer hospitalization days and a greater need for ICU treatment compared to the group without MDs, suggesting a greater burden on the patients and their families. In addition, they had higher maximum mRS scores during the disease and at discharge, indicating they are more severely ill and have a relatively poor short-term prognosis, which is complementary to the results obtained in the ancillary tests. We consider that this might be due to the fact that patients with MDs are prone to combine autonomic dysfunction and manifest disorders of blood pressure regulation, cardiac arrhythmias, and sudden death. When accompanied by decreased level of consciousness, it can lead to prolonged bed rest and induce various complications such as deep vein thrombosis and pulmonary infections. Multiple reasons are likely to result in severe disease and poor prognosis.

In summary, our study found that patients with anti-NMDAR encephalitis with MDs are prone to consciousness disorders, autonomic dysfunction, pulmonary infections, and gastrointestinal dysfunction during the disease. Moreover, they have a more intense inflammatory response, more severe disease, and a relatively poor short-term prognosis. However, there are some shortcomings in our study. First, this study was a single-center, small sample size retrospective study, the results may have limitations, and future prospective studies are needed to expand the sample size and include external data from multiple centers. Second, long-term follow-up was not conducted in this study, which may have an impact on the assessment of prognosis. Finally, we did not explore the clinical characteristics of each subtype of MD, which needs to be further studied.

## 5 Conclusion

In conclusion, this study suggests that MDs in anti-NMDAR encephalitis is associated with multiple complications, serious conditions, and poor prognosis. Early identification and management of MDs should be emphasized in the diagnosis and treatment process and patients with MDs in anti-NMDAR encephalitis should be closely monitored for changes in their condition and timely given symptomatic and supportive treatment.

## Data availability statement

The raw data supporting the conclusions of this article will be made available by the authors, without undue reservation.

## Ethics statement

The studies involving humans were approved by Yunnan First People’s Hospital. The studies were conducted in accordance with the local legislation and institutional requirements. Written informed consent from the patients/participants or patients/participants' legal guardian/next of kin was not required to participate in this study in accordance with the national legislation and the institutional requirements.

## Author contributions

HL: Writing – original draft, Writing – review & editing, JC: Writing – original draft. PZ: Writing – original draft, Writing – original draft. QM: Writing – original draft, Writing – review & editing.
