# Supplementary material for: Analysis of characteristics of movement disorders in patients with anti-N-methyl-D-aspartate receptor encephalitis
Source: Front Neurol. 2024 Mar 1;15:1357697. doi: 10.3389/fneur.2024.1357697 (PMC10941647; doi:10.3389/fneur.2024.1357697)
Supplement: Supplementary file 1 [file Data_Sheet_1.docx]

**Glossary**

| **MDs** | **Definitions** |
| --- | --- |
| OFLD | Involuntary and repetitive movements of the mouth, tongue and face, including frowning and wrinkling of the forehead, sucking, licking, chewing, smacking the lips, sticking out the tongue and curling the tongue, opening and closing the jaws, etc(1). |
| Dystonia | A Consensus Committee was established under the auspices of the Dystonia Medical Research Foundation, the Dystonia Coalition, and the European Dystonia Cooperation in Science and Technology (COST) Action which proposed the following definition of Dystonia: “Dystonia is a movement disorder characterized by sustained or intermittent muscle contractions causing abnormal, often repetitive, movements, postures, or both. Dystonic movements are typically patterned, twisting, and may be tremulous. Dystonia is often initiated or worsened by voluntary action and associated with overflow muscle activation(2).” |
| Stereotypies | Involuntary or unvoluntary, coordinated, patterned, repetitive, rhythmic, purposeless but seemingly purposeful or ritualistic movement, posture or utterance.(3, 4) |
| Myoclonus | Myoclonus is a sequence of repeated, often nonrhythmic, brief shock-like jerks due to sudden involuntary contraction or relaxation of one or more muscles(5). |
| Ataxia | Disorders of co-ordination of movement in the presence of normal muscle strength. Disturbances in the amplitude and coordination of random movements of the limbs, as well as the inability to maintain trunk posture and balance(6). |
| Muscle weakness | Movement disorders in this study were not limited to the spectrum of movement disorders associated with extrapyramidal disease. We defined muscle weakness as the decreased Lovett muscle strength of the patient's limbs, resulting in a limitation of normal movement ability. |
| Tremor | The consensus statement of the movement disorder society on tremor defines it as “Tremor is a rhythmic back-and-forth or oscillating involuntary movement about a joint axis(7).” |
| Catatonia | Catatonia is a complex group of psychomotor syndromes |

**Glossary (continued)**

|  | characterised by a wide range of motor, verbal and behavioural abnormalities. The diagnosis of catatonia is based on the American Diagnostic and Statistical Manual of Mental Disorders, 5th edition, with the presence of at least 3 of the 12 main symptoms: muteness, rigidity, waxing and waning, defiance, stagnation, fixation, agitation, posturing, grimacing, stereotypy, mimicry of speech and mimicry of movement(8). |
| --- | --- |
| Chorea | The ad hoc Committee on Classification of the World Federation of Neurology has defined chorea as "a state of excessive, spontaneous movements, irregularly timed, non-repetitive, randomly distributed and abrupt in character. These movements may vary in severity from restlessness with mild intermittent exaggeration of gesture and expression, fidgeting movements of the hands, unstable dance-like gait to a continuous flow of disabling, violent movements(9). |

Supplementary Table 1 Clinical manifestation and incidence of MDs.

| **MDs** | **Patients (%)** |
| --- | --- |
| OFLD | 33 (67.3) |
| Dystonia | 27 (55.1) |
| Stereotypies | 17 (34.7) |
| Myoclonus | 11 (22.4) |
| Ataxia | 9 (18.4) |
| Muscle weakness | 9 (18.4) |
| Tremor | 5 (10.2) |
| Catatonia | 5 (10.2) |
| Chorea | 5 (10.2) |

MDs, movement disorders; OFLD, orofacial dyskinesia.

Supplementary Table 2 Comparison of the incidence of different MDs in different age groups

| **MDs** | **< 12 years**  **(n=9)** | **12-17 years**  **（n＝12）** | **≥ 18 years**  **（n＝28）** | ***P*** |
| --- | --- | --- | --- | --- |
| OFLD, n (%) | 7 (77.8) | 10 (83.3) | 16 (57.1) | 0.219 |
| Dystonia, n (%) | 4 (44.4) | 10 (83.3) | 13 (46.4) | 0.089 |
| Stereotypies, n (%) | 4 (44.4) | 5 (41.7) | 8 (28.6) | 0.546 |
| Myoclonus, n (%) | 3 (33.3) | 3 (25.0) | 5 (17.9) | 0.586 |
| Ataxia, n (%) | 1 (11.1) | 2 (16.7) | 6 (21.4) | 0.886 |
| Muscle weakness, n (%) | 2 (22.2) | 2 (16.7) | 5 (17.9) | 1.000 |
| Tremor, n (%) | 1 (11.1) | 1 (8.3) | 3 (10.7) | 1.000 |
| Catatonia, n (%) | 0 (0.0) | 0 (0.0) | 5 (17.9) | 0.210 |
| Chorea, n (%) | 4 (44.4) | 0 (0.0) *** | 1 (3.6) *** | 0.003 |

MDs, movement disorders; OFLD, orofacial dyskinesia.

* Means the difference was statistically significant compared to the age <12 years group.

Supplementary Table 3 Comparison of the occurrence of different MDs in different gender groups

| **MDs** | **Female group**  **(n=32)** | **Male group**  **（n＝17）** | ***P*** |
| --- | --- | --- | --- |
| OFLD, n (%) | 20（62.5） | 13（76.5） | 0.321 |
| Dystonia, n (%) | 19（59.4） | 8（47.1） | 0.409 |
| Stereotypies, n (%) | 12（37.5） | 5（29.4） | 0.571 |

Supplementary Table 3 (continued)

| Myoclonus, n (%) | 7（21.9） | 4（23.5） | 1.000 |
| --- | --- | --- | --- |
| Ataxia, n (%) | 5（15.6） | 4（23.5） | 0.770 |
| Muscle weakness, n (%) | 7（21.9） | 2（11.8） | 0.629 |
| Tremor, n (%) | 4（12.5） | 1（5.9） | 0.816 |
| Catatonia, n (%) | 5（15.6） | 0（0.0） | 0.221 |
| Chorea, n (%) | 3（9.4） | 2（11.8） | 1.000 |

MDs, movement disorders; OFLD, orofacial dyskinesia.

**References**

1. Hang H, Lin L, Li D, Li J, Shi J, Lu J. Association between Clinical Factors and Orofacial Dyskinesias in Anti-N-Methyl-D-Aspartate Receptor Encephalitis. *Brain Behav* (2022) 12(7):e2638. Epub 2022/05/28. doi: 10.1002/brb3.2638.

2. Albanese A, Bhatia K, Bressman SB, Delong MR, Fahn S, Fung VS, et al. Phenomenology and Classification of Dystonia: A Consensus Update. *Mov Disord* (2013) 28(7):863-73. Epub 2013/05/08. doi: 10.1002/mds.25475.

3. Varley JA, Webb AJS, Balint B, Fung VSC, Sethi KD, Tijssen MAJ, et al. The movement Disorder Associated with Nmdar Antibody-Encephalitis Is Complex and Characteristic: An Expert Video-Rating Study. *J Neurol Neurosurg Psychiatry* (2019) 90(6):724-6. Epub 2018/07/23. doi: 10.1136/jnnp-2018-318584.

4. Mohammad SS, Fung VS, Grattan-Smith P, Gill D, Pillai S, Ramanathan S, et al. Movement Disorders in Children with Anti-Nmdar Encephalitis and Other Autoimmune Encephalopathies. *Mov Disord* (2014) 29(12):1539-42. Epub 2014/08/27. doi: 10.1002/mds.25999.

5. Sanger TD, Chen D, Fehlings DL, Hallett M, Lang AE, Mink JW, et al. Definition and Classification of Hyperkinetic Movements in Childhood. *Mov Disord* (2010) 25(11):1538-49. Epub 2010/07/01. doi: 10.1002/mds.23088.

6. Dash D, Pandey S. Movement Disorders Associated with Neuronal Antibodies. *Acta Neurol Scand* (2019) 139(2):106-17. Epub 2018/10/20. doi: 10.1111/ane.13039.

7. Deuschl G, Bain P, Brin M. Consensus Statement of the Movement Disorder Society on Tremor. Ad Hoc Scientific Committee. *Mov Disord* (1998) 13 Suppl 3:2-23. Epub 1998/11/25. doi: 10.1002/mds.870131303.

8. Battle DE. Diagnostic and Statistical Manual of Mental Disorders (Dsm). *Codas* (2013) 25(2):191-2. Epub 2014/01/15. doi: 10.1590/s2317-17822013000200017.

9. Barbeau A, Duvoisin RC, Gerstenbrand F, Lakke JP, Marsden CD, Stern G. Classification of Extrapyramidal Disorders. Proposal for an International Classification and Glossary of Terms. *J Neurol Sci* (1981) 51(2):311-27. Epub 1981/08/01. doi: 10.1016/0022-510x(81)90109-x.
